# Flame-Resistant Poly(vinyl alcohol) Composites with Improved Ionic Conductivity

**DOI:** 10.3390/membranes13070636

**Published:** 2023-06-30

**Authors:** Diana Serbezeanu, Corneliu Hamciuc, Tăchiță Vlad-Bubulac, Alina-Mirela Ipate, Gabriela Lisa, Ina Turcan, Marius Andrei Olariu, Ion Anghel, Dana Maria Preda

**Affiliations:** 1 Department of Polycondensation and Thermally Stable Polymers, “Petru Poni” Institute of Macromolecular Chemistry, Grigore Ghica Voda Alley 41A, 700487 Iasi, Romania; chamciuc@icmpp.ro (C.H.); tvladb@icmpp.ro (T.V.-B.); ipate.alina@icmpp.ro (A.-M.I.); 2 Department of Chemical Engineering, Faculty of Chemical Engineering and Environmental Protection, “Gheorghe Asachi” Technical University of Iasi, Bd. Mangeron 73, 700050 Iasi, Romania; gapreot@yahoo.com; 3 Department of Electrical Measurements and Materials, “Gheorghe Asachi” Technical University of Iasi, Bld. Prof. Dr. Doc. D. Mangeron 67, 700050 Iasi, Romania; ina.turcan@yahoo.com (I.T.); molariu@tuiasi.ro (M.A.O.); 4 Academy of Romanian Scientists, Splaiul Independentei 54, 050094 Bucharest, Romania; 5 Police Academy “Alexandru Ioan Cuza”, Fire Officers Faculty, Morarilor Str. 3, Sector 2, 022451 Bucharest, Romania; ion.anghel@academiadepolitie.ro (I.A.); mariadana523@yahoo.com.sg (D.M.P.)

**Keywords:** polyvinyl alcohol, polyphosphonate, flame resistance, dielectric spectroscopy, ionic conductivity

## Abstract

Flame-resistant polymer composites were prepared based on polyvinyl alcohol (PVA) as a polymer matrix and a polyphosphonate as flame retardant. Oxalic acid was used as crosslinking agent. LiClO_4_, BaTiO_3_, and graphene oxide were also incorporated into PVA matrix to increase the ionic conductivity. The obtained film composites were investigated by infrared spectroscopy, scanning electron microscopy, thermogravimetric analysis, differential scanning calorimetry and microscale combustion tests. Incorporating fire retardant (PFRV), BaTiO_3_, and graphene oxide (GO) into a material results in increased resistance to fire when compared to the control sample. A thermogravimetric analysis revealed that, as a general trend, the presence of PFRV and BaTiO_3_ nanoparticles enhances the residue quantity at a temperature of 700 °C from 7.9 wt% to 23.6 wt%. Their dielectric properties were evaluated with Broad Band Dielectric Spectroscopy. The electrical conductivity of the samples was determined and discussed in relation to the LiClO_4_ content. The electrical properties, including permittivity and conductivity, are being enhanced by the use of LiClO_4_. Additionally, a relaxation peak has been observed in the dielectric losses at frequencies exceeding 103 Hz. The electrical properties, including permittivity and conductivity, are being enhanced by the use of LiClO_4_. Additionally, a relaxation peak has been observed in the dielectric losses at frequencies exceeding 103 Hz. Out of the various composites tested, the composite containing 35 wt% of LiClO_4_ exhibits the highest alternating current (AC) conductivity, with a measured value of 2.46 × 10^−3^ S/m. Taking into consideration all the aspects discussed, these improved composites are intended for utilization in the manufacturing of Li-Ion batteries.

## 1. Introduction

The production of batteries with high-energy density and operational safety is increasingly required for application in new advanced technologies. Li-ion batteries had a particular development, as they’re promising in various applications, such as electric vehicles, smart grids, and portable electronics [1]. However, they are limited by safety risks, such as leakage, burning, and even exploding due to the low-boiling point of organic liquid electrolytes. Much research has been undertaken to obtain electrolytes with low flammability and satisfactory ionic conductivity for Li-ion batteries production. Using polymer electrolytes instead of liquid electrolytes has some advantages. Although liquid electrolytes have high ionic conductivity and interact well with battery electrodes, they have some disadvantages, such as high flammability and high vapor pressure at high temperatures. Compared to liquid electrolytes, polymer electrolytes mitigate or impede the growth of metallic dendrites [2], they are more environmentally friendly [3], have a light weight [4], flexibility [4], low electrolyte leakage [5], good processability [6], electrochemical resistance [7], and better fire resistance [8]. They are less reactive with the lithium electrode, and if they exhibit appropriate mechanical performances, they do not require a separator between the electrodes [9].

Poly(vinyl alcohol) (PVA) is a semi-crystalline, water-soluble, environmentally friendly biopolymer that is nowadays produced on a large scale. It has a hydrophilic nature and exhibits good processability, film forming abilities, flexibility, high transparency, non-toxicity, excellent tensile strength, and good thermal stability (200 °C) for applications that do not require higher temperatures for optimal functionality of the material and interesting dielectric properties as was described in certain studies [10,11,12]. However, due to the structure of the chains formed by carbon and hydrogen atoms and the lack of fragments with an aromatic structure, PVA is flammable and has a low thermal stability. Various studies have been conducted to reduce the flammability of PVA [13]. The incorporation of organic phosphorus-containing compounds can substantially improve the flame resistance of PVA without significantly affecting its other properties [13,14,15,16,17]. Fire retardant additives containing phosphorus are usually environmentally friendly with low toxicity, and during combustion, they do not release highly toxic and corrosive products [18,19,20]. Polyphosphonates are a group of phosphorus-containing flame retardants that have been shown to be very effective in increasing the flame resistance of different polymers [21,22,23]. They have good thermal stability, compatibility with PVA, and solubility in organic solvents. Some of them show biodegradability or antimicrobial activity, thus allowing the preparation of materials with superior properties. Polyphosphonates used as flame retardant additives act in the condensed phase to increase the amount of carbonaceous residue that forms a stable protective layer that no longer allows the transfer of heat from the combustion zone to the polymer, nor the removal of combustible gases resulting from polymer pyrolysis. In the gas phase, it forms radicals that inhibit the combustion process [24,25]. 9,10-Dihydro-9-oxa-10-phosphaphenanthrene-10-oxide (DOPO) and its derivatives have the ability to enhance the flame resistance of polymers by incorporating them into the polymer structure. This is attributed to their aromatic and heterocyclic nature, which improves the thermal stability of polyphosphonates and increases the concentration of phosphorus atoms [26,27]. Thus, polyphosphonate-containing DOPO groups have phosphorus atoms in the main and side chain and can be considered to be very effective in improving the thermal stability at high temperatures and reducing the flammability of PVA.

In addition, PVA has been evaluated as a polymer electrolyte for use in obtaining Li-ion batteries [28]. Thus, composite films based on PVA and lithium perchlorate (LiClO_4_) were obtained and characterized. LiClO_4_ exhibits low interfacial resistance when lithium metal was used as anode. Polymer electrolyte is obtained by dispersing LiClO_4_ at a molecular level in PVA. The electrical conductivity of the films as a function of LiClO_4_ concentration in 0.5, 10, 15, and 20 wt%, measured at 750 kHz and room temperature was 9.48 × 10^−5^, 1.78 × 10^−4^, 8.6 × 10^−4^, and 4.80 × 10^−4^ mS/mm, respectively [29]. Solid polymer electrolyte based on PVA and magnesium perchlorate Mg(ClO_4_)_2_ as an electrolytic salt was developed and characterized. The highest electrical conductivity obtained at room temperature was of the order of 10^−4^ S/cm for the sample containing 20% Mg(ClO_4_)_2_ [30]. PVA composites incorporating sodium bromide and silver nanoparticles were prepared, obtaining an ionic conductivity of 1.22 × 10^−4^ S/cm [31]. Polyelectrolytes with a high electrochemical performance can also be prepared by using a mixture of two or more polymers. Thus, polymer composites based on PVA and chitosan were prepared, and their properties were studied. Samples with a concentration of 20% LiClO_4_ showed a conductivity of 3 × 10^−6^ S/cm at room temperature and 8.61 × 10^−5^ S/cm at a temperature of 343 K [32]. Polymer blends based on PVA and polyethylene oxide (PEO), complexed with LiClO_4_ were prepared and investigated. The obtained ionic conductivity was of the order of 10^−3^ S/cm, which is close to that of liquid electrolytes [33]. The introduction of inorganic fillers can improve the room conductivity and mechanical strength of composite polymer electrolyte as well as the interface stability with the electrodes.

The objective of this study is to obtain PVA-based polymer composites with improved characteristics in terms of flame resistance and ionic conductivity for use in the production of Li-Ion batteries. This was achieved by simultaneously incorporating a flame-retardant additive containing phosphorus and LiClO_4_ which was expected to increase the ionic conductivity. The novel PFRV flame retardant has been created by incorporating a greater amount of aromatic groups into its structure, and by adding phosphorus to both the main and side chains. These modifications enable the PFRV additive to work in tandem in both anti-flame gas and solid phase mechanisms, at elevated temperatures, providing enhanced fire resistance properties. Additionally, the combination of crosslinked PVA, PFRV flame retardant, and LiClO_4_ aligns with environmental friendliness. BaTiO_3_ and graphene oxide were chosen as co-additives in order to bring synergistic flame retardant improvements and to see how they influence the electrical conductivity of the PVA-based matrix. The properties of these composites, such as morphology, thermal stability, flame resistance, dielectric characteristics, and ionic conductivity were discussed in correlation with their structure and composition. The composites showed an increase in thermal stability at elevated temperatures and improved flame resistance while maintaining good conductivity at room temperature. In order to improve the physical properties of PVA polyelectrolyte, a crosslinking of the polymer was applied with an introduction in the system oxalic acid as a crosslinking agent.

## 2. Materials and Methods

### 2.1. Materials

Partially hydrolyzed PVA–Mowiol 26–88 (M_w_ = 160,000 g/mol, degree of hydrolysis = 87.7%) was purchased from Zauba (Munchen, Germany). 4,4′-Sulfonyldianiline (DDS), vanillin, LiClO_4_, BaTiO_3,_ graphene-oxide (GO), oxalic acid (OA), tetrahydrofuran (THF), *N,N′*-dimethylformamide (DMF), and phenylphosphonic dichloride were provided from Sigma-Aldrich and used as received. 9,10-Dihydro-9-oxa-10-phosphaphenanthrene-10-oxide (DOPO) was purchased from Chemos GmbH, Altdorf, Lower Bavaria, Germany and dehydrated before use.

### 2.2. Preparation of 4,4′-(Bis(4-hidroxy-3-methoxyphenyl)(6-oxido-6H-dibenzo))

[c,e][1,2]oxaphosphinin-6-yl)methyl)))diaminodiphenylsulfone (PFRV′)

**Scheme 1:**
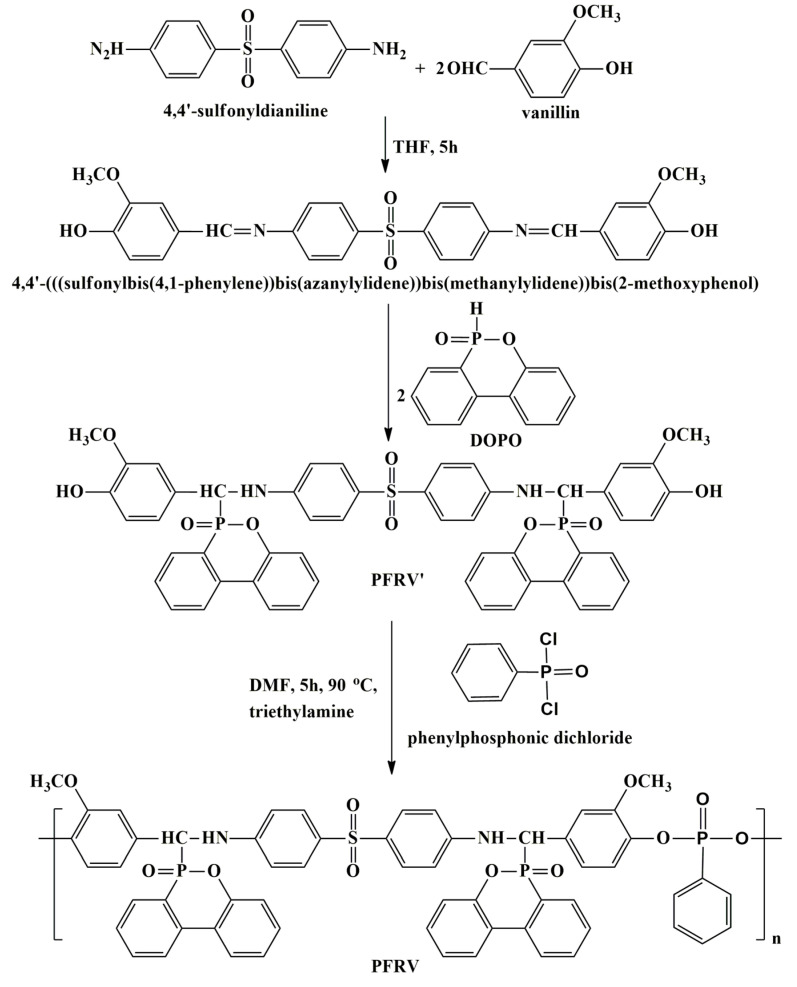
Synthetic pathway to prepare phosphorus-containing polyphosphonate PFRV.

Initially, bisphenol PFRV′ was synthesized using a two-step process carried out in a single reaction vessel that had been modified to include an inlet and outlet for nitrogen atmosphere. In the first step, 4,4′-sulfonyldianiline (4.966 g, 20 mmol) reacted with vanillin (3.043 g, 20 mmol) in THF (60 mL), resulting in a bisphenol containing two imine units. The reaction took place at reflux temperature for 5 h. During the second step of the reaction, an excess amount of DOPO (10.8 g, 50 mmol) was introduced directly into the reaction mixture, and the reflux was continued for 15 h. This led to an addition reaction occurring at the imine bonds, ultimately resulting in the formation of a bisphenol PFRV′ compound that possesses two DOPO side groups (Scheme 1).

The reaction mixture was precipitated in distilled water, and the product was filtered, washed on the filter with distilled water, and dried in a vacuum oven at 80 °C to give a dark yellow product.

Yield = 89%.

FTIR (KBr, cm^−1^): 3369 cm^−1^ (O–H, stretching vibration), 3065 cm^−1^ (aromatic C–H, stretching vibration), 2928 cm^−1^ and 2844 cm^−1^ (aliphatic C–H, asymmetric, and symmetric stretching vibration), 1594 cm^−1^ and 1506 cm^−1^ (aromatic C=C, stretching vibration), 1476 cm^−1^ (aromatic P–C, stretching vibration), 1225 cm^−1^ (aromatic P=O, stretching vibration), 1106 cm^−1^, and 927 cm^−1^ (aromatic P–O–C, asymmetric, and symmetric stretching vibration).

### 2.3. Preparation of Polyphosphonate Based on DOPO and Vanillin (PFRV)

The polyphosphonate PFRV was prepared by reacting bisphenol PFRV′ (4.01 g, 4.23 mmol) with phenylphosphonic dichloride (0.638 mL, 4.5 mmol) in the presence of triethylamine (0.6272 mL, 4.5 mmol) utilized as acid acceptor and DMF (29 mL) as solvent following an adapted method previously reported [34]. The resulting mixture was worked up by precipitation in distilled water. An orange-colored PFRV flame retardant powder was produced by subjecting the precipitate to filtration, washing it with fresh distilled water, and drying in a vacuum oven at 90 °C for 8 h. The reaction sequence for the preparation of polyphosphonate PFRV is also given in Scheme 1.

Yield = 90%.

### 2.4. Preparation of PVA Composites

The PVA composites were obtained with physical mixing of the components listed in Table 1 as described in detail below.

PVA and oxalic acid were dissolved in distilled water under stirring at 60 °C while flame retardant PFRV was dissolved in DMF under stirring at room temperature. To prepare PVA-1 and PVA-2 samples, PFRV was incorporated into PVA by physically mixing them as prepared, corresponding solutions. To manufacture multicomponent film composites (PVA-3–PVA-6), BaTiO_3_, GO, and LiClO_4_ were introduced accordingly into the PVA/OA/PFRV solution followed by homogenizing the mixtures by sonication and advanced magnetic stirring of the resulting suspensions. In each case, the resulting fully homogenized mixtures were cast into Teflon molds and dried, firstly in an ambient condition overnight, then in a vacuum oven at 70 °C for 8 h. Table 1 presents additional data about the preparation of PVA composites.

### 2.5. Measurements

The structure of PFRV was investigated by using FTIR and NMR spectroscopy. The FTIR spectrum was recorded on a FTIR Bruker Vertex 70 Spectrophotometer. The structure of PVA composites was investigated with FTIR spectroscopy using a BioRad ‘FTS 135′ FTIR spectrometer equipped with a Specac “Golden Gate” ATR accessory.

Optical microscopy microphotographs were obtained from all the studied samples at an ambient temperature in order to further investigate their morphology, using a Zeiss Microscope Axio Imager A2M fitted with Linkam Plate LTS420 with a 10× objective (Carl Zeiss Microscopy GmbH, Oberkochen, Germany). Small pieces of dry composite films were cut, placed on glass plates, and examined with the microscopic view set at the edge of the material, before the photos on each sample in the series were taken.

Microscopic investigations of PVA composites and of their corresponding chars were performed on an Environmental Scanning Electron Microscope Type Quanta 200, operating at 10 kV with secondary electrons in low vacuum mode (LFD detector). The Quanta 200 microscope is equipped with an Energy Dispersive X-Ray (EDX) system for qualitative and quantitative analysis and elemental mapping.

Thermogravimetric (TG) curves and thermogravimetric derivative (DTG) curves of PFRV and PVA composites were recorded with a Mettler Toledo TGA-SDTA851^e^ equipment, in a nitrogen atmosphere, and a heating rate of 10 °C/min, in the temperature range of 25–700 °C.

Differential scanning calorimetry (DSC) measurements of PFRV and PVA composites were carried out using a Mettler Toledo DSC1 type device in an inert atmosphere, with a heating rate of 10 °C/min^−1^ and nitrogen purge at 100 mL/min.

The flammability of samples in controlled temperature circumstances was evaluated using microscale combustion calorimetry (MCC) experiments. The tests were carried out in accordance with “Method A”. (ASTM D7309-13).

Broad band dielectric spectroscopy of the samples was performed with a broadband dielectric spectrometer (Novocontrol Technologies, Montabaur, Germany).

## 3. Results

### 3.1. Synthesis and Characterization of PFRV

**Figure 1:**
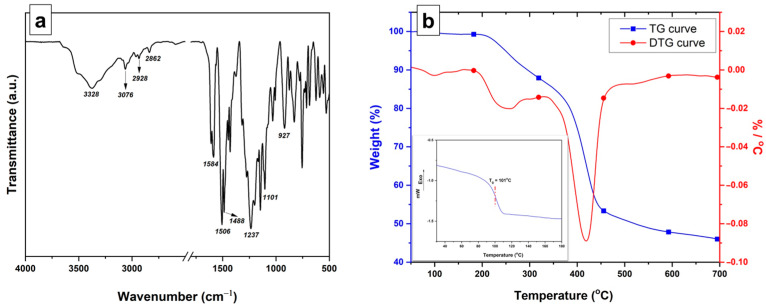
FTIR spectrum (**a**), TG, DTG (**b**), and DSC ((**b**) inset) curves of PFRV.

The chemical structure of PFRV was investigated with FTIR and NMR spectroscopy. The most important absorption bands observed in the FTIR spectrum of the PFRV are at: 3382 cm^−1^ (O–H, stretching vibration), 3076 cm^−1^ (aromatic C–H, stretching vibration), 2928 cm^−1^ and 2862 cm^−1^ (aliphatic C–H, asymmetric and symmetric stretching vibration), 1584 cm^−1^ and 1506 cm^−1^ (aromatic C=C, stretching vibration), 1488 cm^−1^ (aromatic P–C, stretching vibration), 1237 cm^−1^ (aromatic P=O, stretching vibration), 1101 cm^−1^, and 927 cm^−1^ (aromatic P–O–C, asymmetric and symmetric stretching vibration) (Figure 1a). From the ^31^P RMN the following peaks are observed: 31.24, 28.91, −17.39 ppm. The thermal stability of PFRV was evaluated with a thermogravimetric analysis (Table 2, Figure 1). The initial decomposition temperature, considered as the temperature corresponding to the 5 wt% weight loss, *T*_5_, was 244 °C. The thermal decomposition of PFRV took place in two stages. The temperatures of the maximum weight loss rate in the first step and the second step, *T_max_*_1_ and *T_max_*_2_, were 249 and 415 °C, respectively, while the char yield at 700 °C had a value of 45.7 wt%. It can be noticed that in the interval 450–700 °C the weight loss rate of the residue was very low, suggesting a good thermal stability of the char resulted after the pyrolysis process. A glass transition temperature (midpoint of the inflection tangent) of 101 °C was determined from the DSC curve (Figure 1 left down corner inset).

### 3.2. Structural and Morphological Characterization of PVA Composites

**Figure 2:**
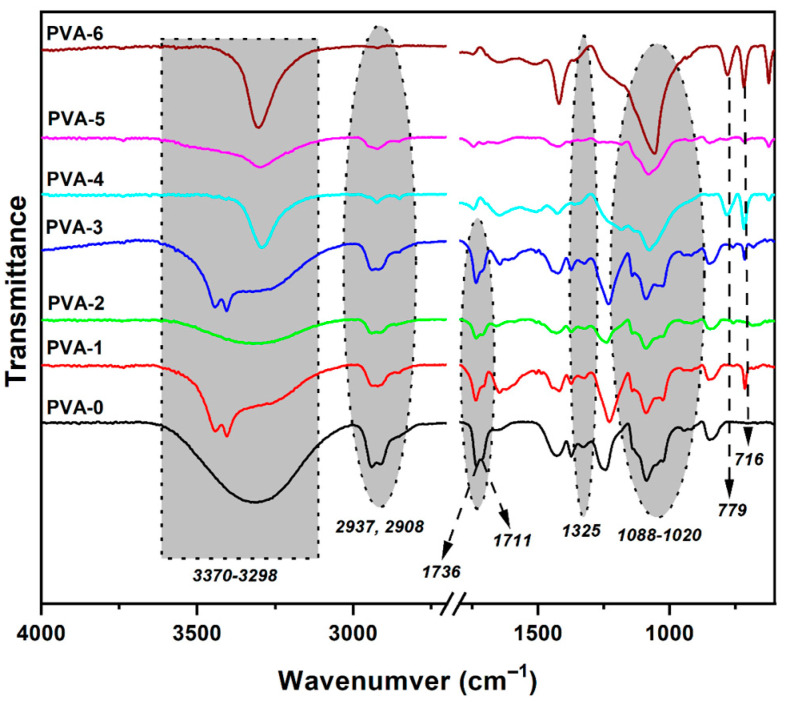
FTIR spectra of PVA composites.

The chemical structure of resulting PVA composite films was investigated by FTIR spectroscopy (Figure 2). Such type of oxalic acid-reinforced PVA composites have been described also in our recent study [35]. The neat PVA-0 showed characteristic absorption bands at 3370 cm^−1^ (O–H stretching vibrations), 2937 and 2908 cm^−1^ (aliphatic C–H asymmetric and symmetrical stretching vibrations), 1736 and 1711 cm^−1^ (belong to residual acetyl groups remaining in fully hydrolyzed PVA), 1424 and 924 cm^−1^ (CH_2_ bending and rocking). The PVA-1–PVA-6 composites exhibited characteristic absorption peaks at 3298 cm^−1^ (O–H stretching vibrations), 2937 and 2908 cm^−1^ (aliphatic C–H stretching vibrations), 1424 cm^−1^ (CH_2_ bending), 937 cm^−1^ (aromatic P–O–C stretching vibrations), 779 cm^−1^ (deformation vibrations caused by the 1,2-disubstituted aromatic phosphaphenanthrene rings), and 716 cm^−1^ (deformation vibrations of the aromatic rings).

The increase of the amount of LiClO_4_ added to the PVA leads to a shift of 72 cm^−1^ of the characteristic absorption of hydroxyl group. The bands at 2937 and 2908 cm^−1^ attributed to asymmetric and symmetric stretching vibration of aliphatic –CH_2_ groups, which become more distinct in PVA composites than in the neat PVA-0. In the case of the PVA-6 the FTIR absorption band observed at 2937 and 2908 cm^−1^, respectively, assigned to C–H stretching vibration, disappeared after LiClO_4_ was added in high amount to the PVA, probably due to formation of PVA crystallites in which the mobility of CH_2_ groups is restrained data observed and for other authors [32,36]. The presence of a sharp band at 1325 cm^−1^ is attributed to the –CH symmetrical deformation mode, specifically the CH_2_ wagging motion. As the concentration of LiClO_4_ increases, this band gradually decreases in intensity. Additionally, a weak band at 1244 cm^−1^, corresponding to the C–H bending vibration, decreases and shifts to 1229 cm^−1^. These observations suggest a strong binding of lithium ions with the PVA composite. Moreover, the broad infrared band at 1024 cm^−1^, associated with the C–O stretching vibration, undergoes a shift to 1046 cm^−1^ in the LiClO_4_^−^ doped composites. The infrared band observed at 838 cm^−1^, which corresponds to the skeletal C–C stretching of pure PVA, undergoes a shift to 779 cm^−1^ when 35 wt% LiClO_4_ (PVA-6) is added. This shift is attributed to the interaction between the charge carriers present in the salt and the C–C group of the PVA host, causing the alteration in the vibrational frequency of the C–C bonds. The infrared band observed at 626 cm^−1^, at higher doping levels, is attributed to the presence of free ClO_4_^−^ anions that do not directly interact with the lithium cations [37,38].

**Figure 3:**
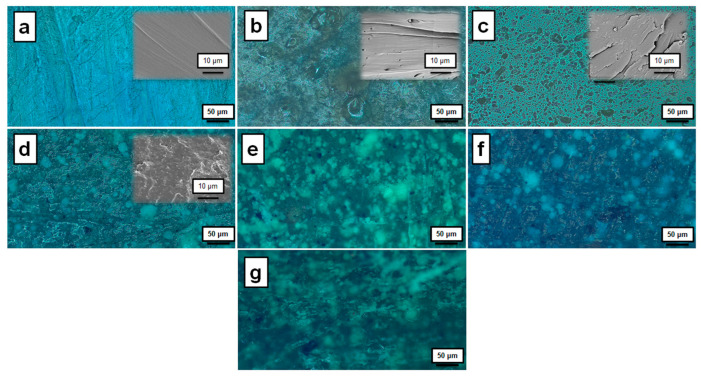
Polarized light microscopy micrographs of the as synthesized PVA composites: PVA-0 (**a**), PVA-1 (**b**), PVA-2 (**c**), PVA-3 (**d**), PVA-4 (**e**), PVA-5 (**f**), PVA-6 (**g**); the insets of the SEM micrographs are given for PVA composites PVA-0 (**a**), PVA-1 (**b**), PVA-2 (**c**), PVA-3 (**d**).

Polarized light microscopy was employed to examine the microscale morphology of the studied PVA composites. In general, the introspected PVA composite films demonstrated morphological variations depending on the type and the amount of the ingredients. In the case of the PVA-0 sample, the pristine PVA macromolecules reinforced with oxalic acid presented a relatively uniform, transparent, and smooth surface without any fracture, and no evident inner channels were observed (Figure 3a). The surface of the film is free from any large bumps or defects, indicating a high degree of purity and homogeneity of the OA-reinforced polymeric matrix, and these findings were also confirmed by imaging the fracture surface of the sample PVA-0 using scanning electron microscopy (Figure 3a inset), revealing a highly homogeneous smooth microstructure.

The specific type and amount of flame retardant used can influence in general the morphology of PVA. Overall, the introduction of PFRV flame retardant significantly impacted the morphology of PVA (Figure 3b,c), suggesting either disrupting mechanisms of the intramolecular (PVA–PVA) and intermolecular (PVA–OA) hydrogen bonding or by affecting the crystallinity of the macromolecular architectures [39]. Thus, the polarized light microscopical introspection revealed a microporous morphology, more pronounced in the case of the sample containing a larger amount of PFRV and also with increasing hole dimensions on sample PVA-2 (Figure 3c) when compared with the PVA-1 sample. Some small defects were present on the fracture surface revealed with an SEM investigation, which also evidenced some more pronounced bumps on the surface of the sample PVA-1 (Figure 3b inset). The frequency and the levels of these bumps were responsible for the increased roughness of the material in the case of sample PVA-2 (Figure 3c inset). Nevertheless, the uniformity of the pore was revealed on the entire microscopic view on sample PVA-2 (Figure 3c).

Furthermore, on continuing complicating the polymer composite systems resulted in more complex morphologies of the composite films upon a closer inspection at the microscale and nanoscale levels. Polarized light microscopy evidenced the acting of BaTiO_3_ (a well-known ferroelectric material) particles as heterogeneous nucleation agents, promoting the formation of some crystalline regions within the PVA–OA polymer matrix. This behavior is responsible for the formation of spherulites, which are characteristic of semi-crystalline polymers and the birefringent appearance of the surface texture upon PLM observation (Figure 3d). In the case of PVA-3 (Figure 3d inset), inorganic BaTiO_3_ nanoparticles embedded on the surface can be observed.

For safety reasons, the SEM images for samples containing LiClO_4_ were not recorded, but, the optical microscopy introspection also evidenced more complex morphologies in the case of samples PVA-4–PVA-6 the ingredients BaTiO_3_, GO, and LiCO_4_ competing the formation of percolative networks of inorganic particles within the polymer matrix. This network governs the development of unique morphologies, such as enlarged and/or interconnected clusters of particles responsible for the increased birefringence of the respective surfaces as can be observed in Figure 3e–g.

**Figure 4:**
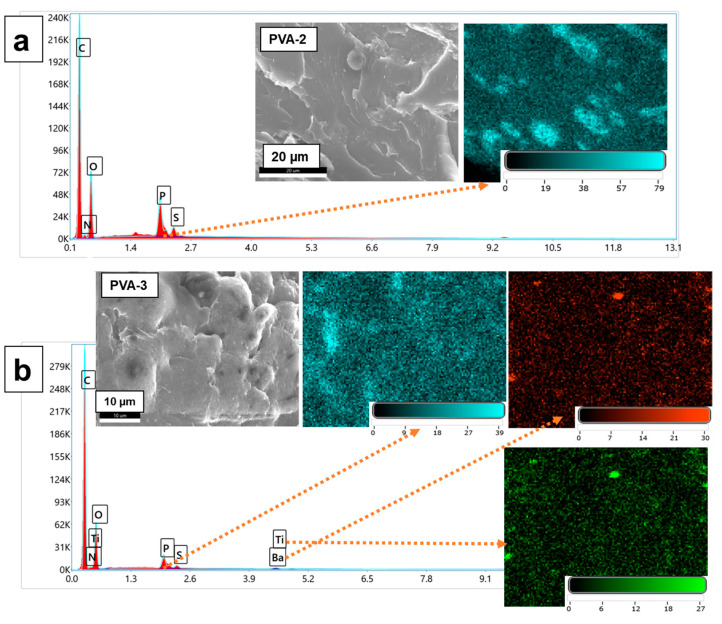
EDX diagram and EDX mapping of PVA-2 (**a**) and EDX diagram and EDX mapping of PVA-3 (**b**).

The confirmation of the presence of certain elements were evidenced further by EDX mapping in the case of two samples as examples in the series. Thus, the EDX map of PVA-2 shows the distribution of nitrogen, phosphorus, and sulfur atoms on the fracture surface. A few small agglomerations of PFRV can be observed on the surface (Figure 4a). EDX mapping of PVA-3 shows the distribution of phosphorus, sulfur, barium, and titanium atoms on the surface. For the most part, the BaTiO_3_ nanoparticles were uniformly distributed (Figure 4b).

### 3.3. Thermal Characterization of PVA Composites

The study of thermal stability and MCC experiments were performed only for the samples denoted as PVA-0, PVA-1, PVA-2, and PVA-3, respectively; the other samples were not investigated for safety reasons, the large amount of LiClO_4_ added into the samples being able to represent a risk in high temperature testing conditions. The thermal stability of the studied samples was evaluated with a dynamic thermogravimetric analysis (TGA). The main determined thermal characteristics are presented in Table 2.

**Figure 5:**
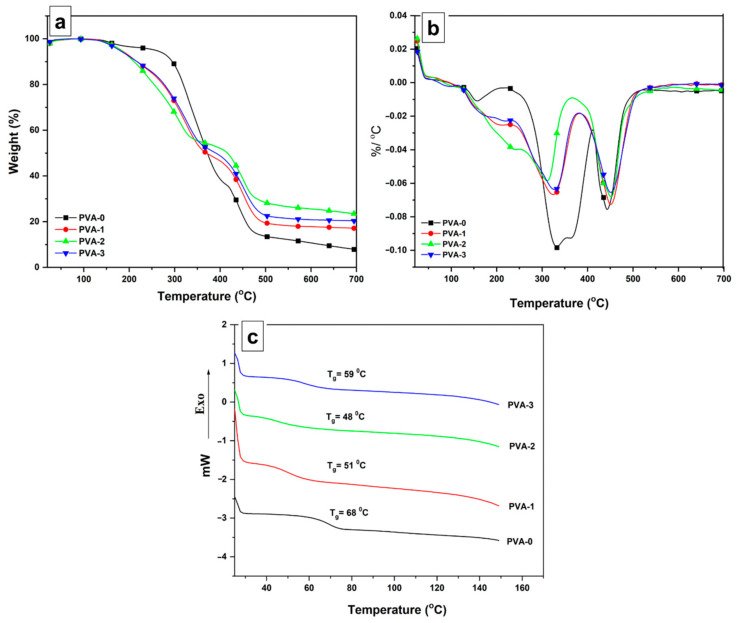
TG (**a**), DTG of the PVA composites (**b**) and DSC curves of PVA composites (**c**).

Figure 5a,b illustrate the TG and DTG curves of the investigated composites. The samples showed an initiation decomposition temperature in the range of 177–251 °C. Compared to the pure PVA-0, a decrease in *T*_5_ values can be observed in the case of the samples containing PFRV flame retardant additive. In addition, the temperature of 30 wt% weight loss decreased by incorporating PFRV into an epoxy resin.

The heat resistance index (*T_HRI_*) was determined using the following relationship [40]:

*T_HRI_* = 0.49[*T*_5_ + 0.6(*T*_30_ − *T*_5_)]

*T_HRI_* quantifies the resistance to a heat flow for a polymer composite. A decrease in *T_HRI_* was observed in the case of the samples containing phosphorus.

The thermal decomposition process took place in two stages. The temperature at which the weight loss rate was maximum in the first stage of decomposition (*T_max_*_1_) was situated in the range of 309–336 °C. *T_max_*_1_ decreased by introducing the PFRV additive and by increasing its content. In the second step of decomposition, the temperature of the maximum weight loss (*T_max_*_2_) was situated in the interval 441–452 °C. A decrease in the maximum weight loss rate in the case of the samples containing PFRV in comparison with the neat sample PVA-0 can be noticed.

The char yield at 700 °C was in the range of 7.9–23.6 wt%. It has increased substantially by the introduction of a PFRV flame retardant additive. For example, the char yield at 700 °C was 17.0 wt% for PVA-1 containing 1% phosphorus while PVA-0 had a value of only 7.9 wt%.

The *T_g_* values of the samples, determined by DSC measurements, were in the interval 48–68 °C. The DSC curves (Figure 5c) revealed a single *T_g_* value for each sample, suggesting good compatibility between epoxy resin and PFRV. A decrease in *T_g_* values appeared in the case of the samples containing phosphorus, probably due to a plasticizing effect of PFRV for epoxy resin.

**Figure 6:**
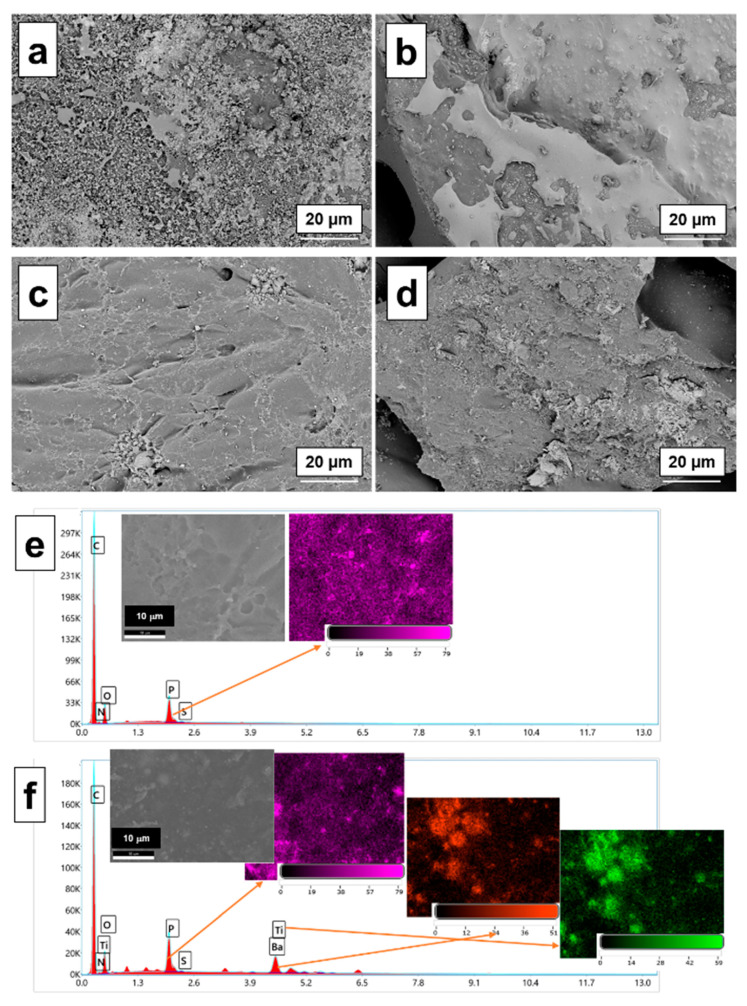
SEM micrographs of PVA-composites heated up to 700 °C PVA-0 (**a**), PVA-1 (**b**), PVA-2 (**c**), PVA-3 (**d**), with the heating rate of 10 °C/min, under nitrogen atmosphere and EDX mapping of PVA-2 (**e**) and PVA-3 chars (**f**) heated up to 700 °C.

SEM images of the chars resulting from heating the samples up to 700 °C with the heating rate of 10 °C/min in nitrogen atmosphere are shown in Figure 6. It can be seen that the carbonaceous residue of PVA-0 showed a large number of pores and holes while those of PVA-1 and PVA-2 were compact and dense. In addition, the residue of PVA-3 exhibited a compact morphology, and BaTiO_3_ nanoparticles were incorporated on the char residue. A mapping technique was used to investigate the atom distribution of the char surface of PVA-2 and PVA-3 (Figure 6e,f). It can be observed the presence of phosphorus, barium, and titanium atoms in higher contents on compared with the corresponding composites. In addition, some agglomerations of these atoms on the fracture surfaces were highlighted.

### 3.4. Microscale Combustion Calorimetry (MCC) Tests

Four composites based on polyvinyl alcohol (PVA-0–PVA-3) were analyzed using microscale combustion calorimetry (MCC). During the MCC tests, the pyrolysis chamber was heated to a temperature of 750 °C using a heating rate of 1 °C/s, and the combustion chamber was maintained at a constant temperature of 900 °C. The combustion properties measured in MCC are related to the flammability characteristics of the material [41,42]. The most relevant data obtained from the MCC tests of the composites are presented in Table 3.

**Figure 7:**
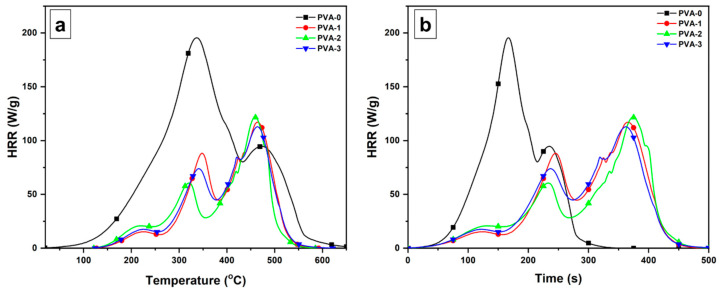
Heat release rates versus temperature for PVA composite samples (**a**) and heat release rates versus time for PVA composite samples (**b**).

In general, the flammability of a material is characterized by the amount of heat released when the material is exposed to combustion (fire) [43]. The dependence of heat release rate (HRR) on temperature and time are presented graphically in Figure 7a,b, respectively. HRR provides information on combustion kinetics and reflects the amount of volatile and flammable compounds. This parameter is often used to assess flammability.

The maximum amount of heat released per unit mass and degrees of temperature, i.e., the HRC (J/g·K) is a material property that appears to be a good indicator of material flammability [44,45]. In this micro-level analysis of the fire performance of building materials using the MCC, low HRC values indicate low flammability in the MCC test and low real-scale fire hazard [46]. Another important parameter is the Char Yield (i.e., the percentage mass of residue left after combustion). Since the percentage of carbon residue is an indication of the amount of unburnt fuel in a material, it is expected that that material with a higher percentage of carbon residue will produce a lower amount of heat during combustion [47]. A correspondence relationship is expected to exist between the two parameters. A low HRC and a high Char Yield are basically indications of a material with increased resistance to burning. For all analyzed samples, a correlation was observed between HRC, THR, and Char Yield.

The lowest HRC and THR values belong to the samples with the highest char yield values, PVA-2 and PVA-3 and the highest HRC and THR values belong to the samples with the lowest char yield values, PVA-0 and PVA-1. The HRC values of the samples were between 326.37 (J/(g·K)) for the PVA-0 and 231.1 (J/(g·K)) for the PVA-3. The THR values are between 20.06 (kJ/g) for the control PVA-0 and 16.86 (kJ/g) for the PVA-2 sample, followed in increasing order by 18.04 (kJ/g) for sample PVA-3 and 18.30 (kJ/g) for sample PVA-1. In terms of THR, similar results are observed between PVA-1 and PVA-3. The char yield values in descending order were as follows: 13.97% for sample PVA-2, 12.04% for sample PVA-3, 8.50% for sample PVA-1, and 3.12% for sample PVA-0. Thus, the addition of BaTiO_3_ and graphene oxide brings benefits from the point of view of these parameters.

Analyzing Figure 7a, the PVA-0 showed two peaks at different PHRR values of 195.57 W/g at 388.09 °C and 94.71 W/g at 456.32 °C, respectively. The composite samples PVA-1, PVA-2, and PVA-3 each show 4 peaks with PHRR values that fall in the range of 15.26–121.73 W/g and appear around the following temperatures: 225, 337, 421, and 462 °C, respectively. It is obvious that the highest peak of PVA-0 was at 388.09 °C, and compared to it, lower values of PHRR corresponding to composite samples PVA-1–PVA-3 were observed, so the PHRR of composite samples decreases by about 35% compared to the control evidence.

Figure 7b shows the effect of the addition of a flame retardant, phosphorus, BaTiO_3_, and graphene oxide (GO) in delaying the onset of PHRR by 72 s of the composite samples compared to the control sample.

From the analyzed data, it follows that by adding a fire retardant (PFRV), BaTiO_3_, and graphene oxide (GO), materials with increased resistance to the action of fire are obtained compared to the control sample. The best results are observed for PVA-2 and PVA-3, which have in their composition phosphorus in higher concentrations and, respectively, the mentioned additives. These samples show the best thermal stability among the composite samples, which means that they produce a lower quantity of combustible substances than the PVA-0 control sample, so they are less flammable.

### 3.5. Electrical Properties

Regarding the behavior of electrical properties at room temperature, a clear difference can be observed between the characteristics of the composites containing LiClO_4_ (PVA-4, PVA-5, and PVA-6) and those without LiClO_4_.

**Figure 8:**
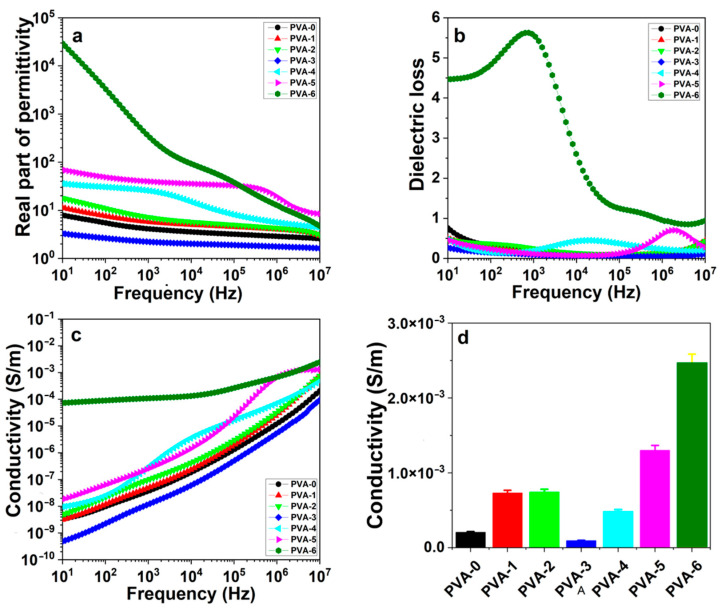
Frequency dependence of the: real part of permittivity (**a**), dielectric loss (**b**), and conductivity at room temperature for PVA-composites (**c**). The electrical conductivity of PVA-composites measured at frequency of 10 MHz (**d**).

The real permittivity of the reference sample, PVA-0, is quite independent on frequency as it can be noticed from Figure 8a. A visible but not important increase in the real permittivity at lower frequencies can be observed for PVA-0 fact, which can be attributed to interfacial polarization, which is occurring while varying the frequency. This small increase is also noticeable for the dielectric losses at frequencies below 102 Hz. While increasing the quantity of PFRV and P (samples PVA-1 and PVA-2), an insignificant increase in the real permittivity occurs, but its dependence on frequency is still negligible.

The addition of BaTiO_3_ and GO (sample PVA-3) is leading to a decrease of the real permittivity and of the losses. The utilization of LiClO_4_ (sample PVA-4, PVA-5, and PVA-6) is leading to an increase in the electrical properties (both permittivity and conductivity) and the occurrence of relaxation peak in the dielectric losses at frequencies above 103 Hz (Figure 8b). The increase of the LiClO_4_ content in the case of PVA-6 in comparison to PVA-4 and PVA-5 is leading to a more visible interfacial polarization fact, which is emphasized by the increase at lower frequencies of real permittivity and appearance of a protuberant relaxation. Such behavior is usually attributed to extrinsic phenomena, represented by slow charge species (space charge effects), such as electron hopping, oxygen vacancies, and boundary defects. However, this behavior in respect to electrical properties, mostly at lower frequencies, may be attributed to LiClO_4_ as the elimination of PFRV and P from the sample (sample PVA-5), which is leading to an increase in the real permittivity, and a displacement of the relaxation peak on dielectric losses curve towards higher frequencies. The addition of LiClO_4_ with the polymer matrix may result from the localization of charge carriers along with mobile ions, thereby causing higher ion conductivity [48].

The variation of AC conductivity with a frequency for all polymer electrolytes at room temperature is depicted in Figure 8c. The samples without LiClO_4_ display similar patterns for conduction spectra while the LiClO_4_-based composites exhibit a rather complex behavior with a large frequency-independent plateau for PVA-6 sample, indicating the contribution from the DC conductivity. The LiClO_4_-based PVA composites show an increase in ionic conductivity with increasing salt as well as frequency, and the highest ac conductivity of 2.46 × 10^−3^ S/m is observed for PVA-6 containing 35 wt% of LiClO_4_ (Figure 8d). This is attributed to the interaction of Li^+^ ions with OH groups of PVA, which forms the charge transfer complexes and acts as hoping site for Li^+^ ions to migrate into PVA composite, and the barrier between the capture sites is expected to be reduced in order to create a path through the amorphous region of the polymer matrix, thus enhancing the conductivity [32].

**Figure 9:**
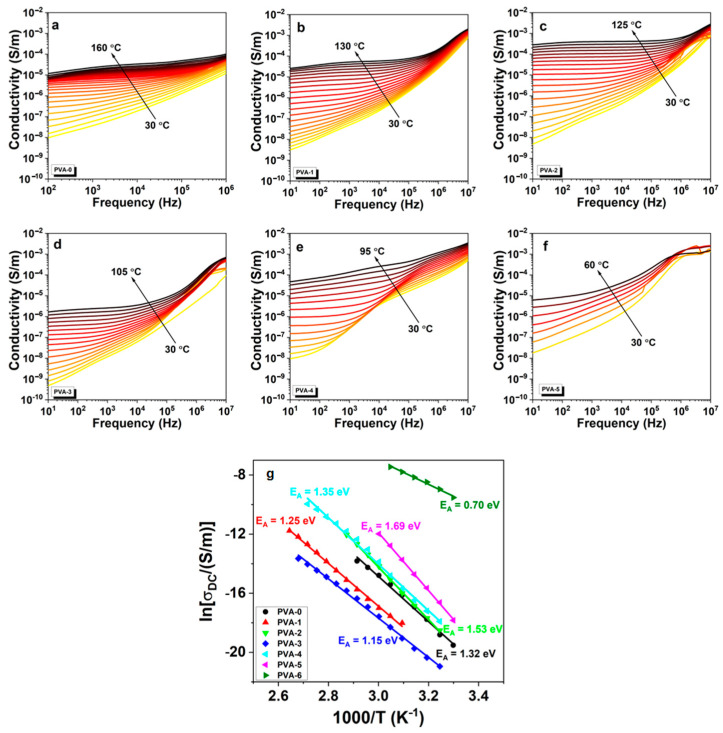
Frequency-dependent variation of the AC conductivity of PVA-composites measured at different temperatures (**a**–**f**). Arrhenius plots and the extracted activation energy values (**g**).

In order to understand the relaxation phenomena of these polymer composites as a function of the substituent components and their relationship with possible ionic defects, a complex analysis of conductivity was performed. This analysis provides significant information related to the transport of charge carriers (i.e., electrons/holes or cations/anions) dominating the conduction process and their response as a function of temperature and frequency. Figure 9a–g show the variation of ac conductivity with frequency at different temperatures for all investigated PVA composites. If at low temperatures the conductivity showed dispersion in frequency (the conductivity increased with increasing frequency), at higher temperatures the conductivity spectra were characterized by the appearance of a frequency-independent plateau (the appearance of DC conductivity). In this plateau region, the conductivity ($\sigma_{DC}$) increased with temperature, showing the thermally activated semiconductor behavior of the conduction process. Therefore, as in the case of ionic solids, the thermally activated DC conductivity is described by the Arrhenius law:

$$\sigma_{DC}=\sigma_{0}e^{-\frac{E_{A}}{kT}}$$

where $\sigma_{0}$ is a pre-exponent, $E_{A}$ is the activation energy, and k is the Boltzmann constant.

In the literature, activation energy was defined as the minimum energy uses for the migration of ions inside the polymer electrolyte system [49]. Referring to Figure 9g, PVA-6 has the lowest $E_{A}=0.7$ eV value, which signifies that this sample has the smallest energy barrier for the migration of Li^+^ ions. Activation energies of the order of 0.7–1.35 eV were also identified in the case of polymer composites PVDF:PVA:NH_4_NO_3_ [50].

## 4. Conclusions

The primary aim of this paper was to create advanced composite films using PVA that could be utilized in high-performance technologies. This was achieved by modifying the thermal, flame retardant, and electrical properties of pure PVA through the use of various components and additives, resulting in a more innovative material. A novel polyphosphonate based on vanillin and PFRV and containing phosphorus in both main and side has been developed and utilized as an additive to impart flame retardancy while BaTiO_3_, GO, and LiClO_4_ nanoparticles were introduced into the multicomponent PVA-OA-based composite films used to modulate the electrical performances of the finite materials. Thermogravimetric analysis indicated that in general the introduction of PFRV and BaTiO_3_ nanoparticles increases the amount of residue at 700 °C, while the introduction of BaTiO_3_ nanoparticles into the system increased further this parameter. The MCC test indicated a lower flammability in the case of the samples incorporating PFRV and BaTiO_3_ nanoparticles. The inclusion of LiClO_4_ (found in samples PVA-4, PVA-5, and PVA-6) is causing a rise in the electrical characteristics like permittivity and conductivity. Additionally, a relaxation peak in the dielectric losses at frequencies higher than 103 Hz was noticed. The PVA composites containing LiClO_4_ exhibited a rise in ionic conductivity as both the salt concentration and frequency increase. Among them, PVA-6 with 35 wt% of LiClO_4_ displays the highest AC conductivity, measured at 2.46 × 10^−3^ S/m.

## Figures and Tables

**Table 1:** Detailed data regarding the preparation of PVA composites.

Sample	PVA (g)	PFRV (g/%)	P (%)	BaTiO_3_ (g/%)	GO (g/%)	LiClO_4_ (g/%)	OA (g/%)
PVA-0	2.15	0	0	0	0	0	0.162
PVA-1	2.15	0.30/11.48	1	0	0	0	0.162
PVA-2	2.15	0.690/22.25	2	0	0	0	0.162
PVA-3	2.15	0.319/11.48	1	0.138/5	0.0083/0.3	0	0.162
PVA-4	2.15	0.298	1	0.109/5	0.0065/0.3	0.64/20	0.162
PVA-5	2.15	0	0	0.109/5	0.0065/0.3	0.5663/20	0.162
PVA-6	2.15	0.298	1	0.109/5	0.0065/0.3	1.38/35	0.162

**Table 2:** Thermal properties of PFRV and PVA composites.

Sample	*T_g_*^(1)^ (°C)	*T*_5_^(2)^ (°C)	*T*_30_^(3)^ (°C)	*T_HRI_*^(4)^ (°C)	*T_max_*_1_^(5)^ (°C)	*T_max_*_2_^(5)^ (°C)	Char Yield at 700 °C (wt%)
PFRV	101	244	402	166	249	415	45.7
PVA-0	68	251	339	149	336	441	7.9
PVA-1	51	184	308	125	324	452	17.0
PVA-2	48	182	293	122	309	450	20.5
PVA-3	59	177	314	127	327	451	23.6

^(1)^ Glass transition temperature; ^(2)^ Temperature at 5 wt% wight loss; ^(3)^ Temperature at 30 wt% wight loss; ^(4)^ The heat resistance index; ^(5)^ Temperature at which the mass loss is the highest in the two decomposition stages.

**Table 3:** Data obtained by MCC analysis for the PVA composites.

Samples	PVA-0	PVA-1	PVA-2	PVA-3
CY (wt%)	3.1	8.5	13.9	12.0
THR (kJ/g)	20.6	18.3	16.8	18.0
PHRR 1 (W/g)	195.6	15.2	20.8	17.5
T_PHRR 1_ (°C)	388.1	229.9	220.2	224.7
Time 1 (s)	167.0	128.0	132.5	121.5
PHRR 2 (W/g)	94.7	87.2	60.5	73.9
T_PHRR 2_ (°C)	456.3	349.8	321.1	340.4
Time 2 (s)	234.5	247.0	233.5	236.5
PHRR 3 (W/g)	-	84.0	70.9	84.6
T_PHRR 3_ (°C)	-	424.1	418.1	420.5
Time 3 (s)	-	325	334	319
PHRR 4 (W/g)	-	117.1	121.7	112.9
T_PHRR 4_ (°C)	-	463.4	458.5	463.5
Time 4 (s)	-	365	374	362
HRC (J/(g·K))	326.3	321.3	231.3	231.1

CY = char yield; PHRR = Heat release peak; THR = Total heat release; T_PHRR_ = Temperature of heat release peak; Time = The time to attain heat release peak; HRC = Heat release capacity.

## Data Availability

Not applicable.
